# Today’s Mediterranean Diet in Greece: Findings from the National Health and Nutrition Survey—HYDRIA (2013–2014)

**DOI:** 10.3390/nu14061193

**Published:** 2022-03-11

**Authors:** Georgia Martimianaki, Eleni Peppa, Elisavet Valanou, Eleni M. Papatesta, Eleni Klinaki, Antonia Trichopoulou

**Affiliations:** Hellenic Health Foundation, HHF, Kaisareias 13 & Alexandroupoleos, 115 27 Athens, Greece; e.peppa@hhf-greece.gr (E.P.); valanou@hhf-greece.gr (E.V.); m.papatesta@gmail.com (E.M.P.); eleni.t.klinaki@gmail.com (E.K.); atrichopoulou@hhf-greece.gr (A.T.)

**Keywords:** national survey, nutrition, Greece, Mediterranean diet, dietary guidelines, representative, dietary intake, 24 h recall, macronutrients, public health

## Abstract

Background: This study aimed to investigate the food and macronutrient intake of the population in Greece and evaluate its adherence to the Greek traditional Mediterranean diet. Methods: Adults over 18 years old (*n* = 4011) were included from the 2013–2014 National Health and Nutrition survey—HYDRIA. Dietary intake was collected using two 24-h recall interviews and a nonquantitative food frequency questionnaire. Macronutrient intakes were calculated using an updated version of the Greek FCT. Results: Only 28.3% of the adult population had high adherence to the Greek traditional Mediterranean diet, with a higher percentage (39.7%) observed for participants over 65 years compared to those under 65 years (25.5%). Differences in adherence to the MD were observed among the four geographical regions in Greece. Younger adults had a higher intake of meat, cereals, alcoholic and nonalcoholic beverages, and sugar products than older individuals who consumed more vegetables, fruits, legumes, dairy, fish, and lipids (mainly from olive oil). Adults do not meet the international dietary recommendations for the intake of several foods and macronutrients. Conclusions: The adult Greek population, especially younger people, has headed away from the Greek traditional Mediterranean diet. These observations indicate potential detrimental consequences in terms of morbidity and mortality.

## 1. Introduction

National health and nutrition surveys are designed to assess the health and nutritional status of a population and are considered important. The World Health Organization (WHO) encourages countries to collect dietary data at an individual level using nationally representative health and nutrition surveys [1]. A review conducted in 2017 [2] reported that less than two-thirds of the European countries have conducted national representative nutrition surveys, a number smaller than the number of nutrition surveys (and their follow-ups) conducted in other countries outside Europe. Health and nutrition surveys are internationally recognized as an important public health indicator for monitoring the nutritional status and health of populations. In Greece, since 2010, three nationally representative studies have been conducted, the HYDRIA (2013–2014) [3,4] survey, the PA.ME.D.Y (2013–2015) [5,6,7], and the EMENO (2013–2016) [8,9] study. PA.ME.DY investigated the association of dietary patterns with health and lifestyle characteristics of the adult Greek population [5,6], while EMENO investigated the morbidity and cardiovascular risk factors of the adult population [9,10]. The HYDRIA survey in the review of Rippin et al. was cited as the only national survey in Greece that collected food, macronutrient, and micronutrient intakes in a representative sample of adults [2]. Furthermore, HYDRIA is the only Greek study whose methodology has been approved by the European Food Safety Authority (EFSA) and has been further adapted in two new studies: the EFSA Support on 780 individuals aged 10 to 74 years old in the context of the pan-European Food Consumption Survey, “What’s on the Menu in Europe?” (EU Menu) [11], and the Pilot HYDRIA-children on children aged 3 months to 10 years in Greece [12].

The nutritional status of population can be assessed either through the estimation of individual food and nutrient intakes and/or through the adherence to dietary patterns. The last study assessed of the changes in the nutritional behaviour of Greeks at a population level was the Greek EPIC (European Prospective Investigation into Cancer and Nutrition) cohort, which was held for 14 years (1997–2011). The findings of 23,505 participants reported an increase in the consumption of vegetables, fruits, and fish/seafood over the years, and a decrease in dairy products, nuts, cereals, and meat consumption, while no prevailing trend was found or observed for legume consumption [13]. Some studies have also investigated the adherence of subpopulations to dietary patterns in Greece, with emphasis given to the Mediterranean diet (MD) [14,15,16]. The MD pattern includes the dietary habits traditionally followed by the populations living around the Mediterranean Sea, with numerous well-established benefits for the health of the inhabitants. In brief, the Greek traditional MD is characterized by high intakes of fruits and nuts, vegetables, legumes, cereals, and olive oil; moderate intakes of fish, dairy products (mainly yoghurt and cheese), and alcohol (especially wine during meals); and a low intake of meat [17].

To the best of our knowledge, there are no other studies that described the usual food consumption for a large variety of food groups and nutrients and that are directly comparable to those of other European countries, as well as the adherence to the MD in a representative sample of the adult population in Greece. The HYDRIA survey collected representative comparable data across EU Member States using standardized procedures in order to provide conclusions and useful observations on population health and related key risk factors, such as the nutritional status of a population as a whole or of specific population groups, and at the same time to fill the gap in providing data on reports requested by international organizations. The present paper aims to describe the dietary habits, including usual energy, food, and macronutrient intakes, of adult men and women living in Greece and the population’s adherence to the Greek traditional Mediterranean diet overall and by age groups.

## 2. Materials and Methods

The study design for the HYDRIA survey followed the recommendations of the European Health Examination Survey (EHES) [18] and the European Food Safety Authority (EFSA) [19] for the collection of health and dietary data at a national level, allowing for intercountry comparisons. It was conducted according to the guidelines in the Helsinki declaration and national legislation on the protection of individual data. All individuals signed informed consent prior to the study initiation.

Eligible individuals were men and women aged 18 years and over residing permanently in Greece based on the latest Greek general census (2011). A two-stage stratified random sampling scheme was applied, with the primary sampling unit being the municipality/local community (first stage) and the final unit being the individual (second stage) for to select the HYDRIA representative survey sample. The HYDRIA survey sampling frame covered all the 51 prefectures of the 13 regions of the country [3].

The data collection included interviewer-administered questionnaires on sociodemographic and lifestyle characteristics, and medical history, while measurements of blood pressure, somatometric characteristics, and blood samples were collected by health professionals. Study centres, mainly including health care facilities close to the residences of individuals, were used for the data collection. The data collection started in June 2013 and was completed in December 2014. Overall, 1873 men and 2138 women, aged 18 to 94 years old, participated in the HYDRIA survey, with a 50% response rate. Details for the design, methodology, and data collection of the HYDRIA survey have been described elsewhere [3,4].

Dietary intake was collected using two nonconsecutive 24 h dietary recalls (24-HDR) per participant and a nonquantitative food frequency questionnaire (food propensity questionnaire, FPQ). The two 24-HDRs were distributed over seasons (in group level) and days of the week to control variations in food consumption. The first 24-HDR (face-to-face interview) was carried out on the first day of the participants’ examination, while the second 24-HDR (by telephone) was performed approximately 15 to 30 days after the first recall. During a 24-HDR, participants were asked to report in detail all the food and beverages they had consumed the previous day. In addition, questions were asked about food preparation practices, types of foods (e.g., type of fat used during cooking, type of milk), and the amounts consumed. The 24-HDR data were collected by trained interviewers using a specially designed application, the HHF Nutrition Tool. The HHF Nutrition Tool included a food list, facets, and descriptors of food items that were in line with both the LanguaL™ Food Description Thesaurus [20] and EFSA’s FoodEx2 classification system. A validated food photography atlas with pictures of 170 food items and recipes in different portions, standard portions sizes of specific foods, and household measures were used to help participants quantify the food, drinks, and dishes consumed [21].

In addition, participants were asked to report the frequency of consumption (never, less than 1 day per month, 1–3 days per month, 1 day per week, 2–3 days per week, 4–5 days per week, 6–7 days per week) of eighty-eight foods and dietary supplements in the previous year through an interviewer-administered, nonquantitative food frequency questionnaire (FPQ). The FPQ for the HYDRIA survey was developed and pilot-tested in the context of the PANEU project [22].

All the collected data are inputted and stored electronically on the HHF central server anonymously and securely to protect participants’ privacy. The HYDRIA survey data are protected under applicable national law and by approval from the Hellenic Data Protection Authority (HDPA, www.dpa.gr, accessed on 9 January 2022) for the establishment and operation of a file registry of sensitive personal data under law N.2472/1997.

### Statistical Analysis

Individuals who did not participate in any of the two 24-HDRs (*n* = 60) were excluded from the analyses. For calculating the energy intake and macronutrients, the foods consumed were linked through the HHF nutrition tool to the HYDRIA Food Composition Table (H-FCT), which was created to accommodate the recorded consumption data. For this purpose, the existing FCT [23,24] was enriched with more food items, and nutrients were further expanded and updated. Under-reporters in the HYDRIA survey were identified as 31% of men and 35% of women based on Schofield et al. and Golberg et al. equations [25,26,27]. For the calculation of under-reporters, we assumed a light physical activity level equal to 1.55 METs (based on metabolic equivalents) [28], considering that more than 60% of participants reported a light physical activity in the HYDRIA survey. Under-reporters were not excluded from the present analyses because their exclusion could include bias [29].

In the present analysis, we estimated the intake of energy and macronutrients (protein, glycaemic carbohydrates, dietary fibre, total fat, saturated fatty acids (SFA), monounsaturated fatty acids (MUFA), polyunsaturated fatty acids (PUFA), and alcohol) for the following thirteen food groups: vegetables (excluding potatoes), potatoes, legumes, fruits (without fresh fruit juices), cereals (including cereal products), dairy (including dairy products), meat (including meat products), fish (including seafood and fish products), eggs, fats and oils, nonalcoholic beverages, sugar and products, and alcoholic beverages, and eleven food subgroups: bread, pasta, rice, milk, yoghurt, cheese, poultry, red meat, olive oil, wine, and beer.

The usual mean intake of energy, food groups and subgroups, and macronutrients was estimated using the mean of the two individual corresponding 24-HDRs under the assumption that two or more 24-HDRs per person give unbiased estimates of the single-day intakes [30]. The usual mean intake of food items and macronutrients was further energy-adjusted per 2000 kcal/day and expressed as a percentage contribution to the total energy intake (%EI), respectively, to evaluate their contribution to a diet with the same total energy intake and to compare subpopulations with different energy intakes.

To estimate the distribution (median, 25th, and 75th percentiles) of the usual intake of energy, food groups and subgroups, and macronutrients, we used the statistical method published by the National Cancer Institute (NCI, Betsheda, MD, USA) [30,31,32]. The NCI method addresses biased estimates of other distribution values except for the mean due to the problem of within-individual variability from day-to-day variation in diet and other random sources of measurement error [30]. This method uses mixed-effects models to estimate the distribution of usual intake of single dietary components consumed on an almost daily basis by nearly everyone, such as nutrients, or episodically consumed components, such as foods, including a large proportion of zero intakes on any given day. A two-part model (considering the probability of consuming food on a particular day and the amount eaten on the consumption day) for repeated measures data with correlated random effects was used to estimate the usual intake distributions of all food items and alcohol, while a one-part model (considering only the amount eaten on a given day) was used to estimate the usual intake distributions of energy and macronutrients.

The adherence to the Greek traditional Mediterranean diet was assessed with a nine-point score for the consumption of vegetables, legumes, fruit and nuts, cereal, fish, meat, dairy, alcohol, and lipids (expressed as the ratio of monounsaturated to saturated lipids [33]) incorporated in a mixed-effects model developed by the NCI for the assessment of multicomponent dietary data [34,35]. More specifically, using the sex-specific median, a value of 0 was given to participants whose consumption of vegetables, legumes, fruits and nuts, cereals, and fish was below the median consumption, while a value of 1 was assigned in the opposite case. Study participants with a below-median consumption of meat and dairy products were assigned a value of 1, whereas a value of 0 was given to those with an above-median consumption. For ethanol, men consuming 10–50 g/day and women consuming 5–25 g/day were given a value of 1 [33]. The Mediterranean diet score was then categorized into three categories: low adherence, including individuals with a total score of 0 to 3 points; intermediate adherence, with a score of 4 to 5 points; and high adherence, with a score of 6 to 9 points [33].

To estimate the distribution of usual dietary intakes and Mediterranean diet score, all models were stratified by gender and included covariates for ten-year age groups (18–24 years, 25–34 years, to 75 years and older), education level (low, intermediate, high), and geographic area (Attiki, Northern Greece, Central Greece, and the Aegean islands and Crete). In addition to the estimation of the usual distribution intake of food groups and subgroups, the corresponding frequency food variables of FPQ were included in the models to supplement the food intake obtained from the two 24-HDRs. Weighting factors were applied in all models to achieve nationally representative results in the usual intakes. The weighting factors were calculated using the 2011 Greek census, considering the complex sampling study design and the response rate of participants by sex, age group, geographical region, and urbanization level.

## 3. Results

Table 1 shows selected sociodemographic and lifestyle characteristics of the participants in the HYDRIA survey. Of note, in the study sample, 48.1% were men and 51.9% were women. The majority of participants were between 18 and 64 years old, with 77% men and 73.1% of women, versus 23% of men and 26.9% of women over 65 years old. Men reported having an intermediate level of education (41.1%), being employed (52%), having a moderate-intensity level of physical activity (3.7%), being more likely to be current smokers (39.4%), and being overweight (43.4%), while women reported having a low education level (44.1%), having a light-intensity level of physical activity (80.5%), being more likely to be never-smokers (54.8%), and being obese (35.6%).

### 3.1. Energy Intake

Table 2 shows the mean, median, 25th, and 75th percentiles of the usual daily energy intake of adult individuals in Greece. Mean and median energy intakes were 2192 kcal/day and 2109 kcal/day in men, while in women were 1530 kcal/day and 1473 kcal/day, respectively. Energy intake was reported lower among older men and women. After excluding the under-reporters, the mean energy intake was 374 kcal/day and 318 kcal/day higher in men and women, respectively.

### 3.2. Macronutrients Intake

Table 3 shows the percentage of contribution of macronutrients to the total energy intake (%EI) and the distribution of their usual daily intake by sex and age group. Overall, the contribution of protein, total fat, MUFA, SFA, PUFA, and alcohol to the total energy intake was 15.1%, 42.3%, 20.3%, 13.1%, 5.7%, 5.4% in men, and 15.2%, 42.9%, 20.6%, 13.5%, 5.9% and 1.8% in women, respectively. The %EI from total carbohydrate consumption, including glycaemic carbohydrate and dietary fibre, was 36.9% (35.2% for glycaemic carbohydrate, 1.7% for dietary fibre) in men and 40.1% (38.2% for glycaemic carbohydrate, 1.9% for dietary fibre) in women. Older individuals (above 65 years old) had a higher MUFA % energy intake than younger individuals (21.9–21.3% versus 20.1% of EI). The distribution of the usual intake of macronutrients intake, including median, 25th, and 75th percentiles, was higher in men and women below 65 years old compared to those above 65 years old.

### 3.3. Foods Intake

Table 4 shows the mean energy-adjusted intake and the distribution of the usual daily intake of food groups and selected subgroups by sex and age group. Considering the usual mean intakes in a diet of 2000 kcal/day, men and women over 65 years old consumed less meat and more fruit, vegetables, fish, and olive oil than those under 65 years old. Similar were the results for the distribution values of the usual intakes. Older men and women had higher median intakes of vegetables (m: 208 g/day, w: 161 g/day), fruits (m: 119 g/day, w: 133 g/day), legumes (m: 22 g/day, w: 11 g/day), and fish (m: 19 g/day, w: 20 g/day). Younger men and women consumed more meat (m:108 g/day, w: 64 g/day), cereals (m: 240 g/day, w: 159 g/day), nonalcoholic beverages (m: 136 g/day, w: 73 g/day), alcoholic beverages (m: 114 g/day, w: 32 g/day), fats and oils (m:46 g/day, w: 32 g/day), and sugar products (m: 25 g/day, w: 28 g/day). Among the main food groups (meat, cereals, dairy, and alcoholic beverages), the food subgroups of bread, pasta, red meat, cheese, wine, and beer were preferred by younger individuals, while yoghurt and milk were preferred by older adults. Men and women below 65 years had a median olive oil consumption of 35 g/day and 25 g/day, respectively, while the intakes of older men and women were 32 g/day and 24 g/day, respectively. When we compared the usual median intakes between sexes, men <65 years consumed more vegetables, potatoes, legumes, cereals, meat, fish, egg, lipids, and nonalcoholic and alcoholic beverages compared to women who consumed more fruits, dairy, and sugar products. The distribution of results was similar in men and women ≥ 65 years, including higher intakes of dairy and fish in men and women, respectively.

### 3.4. Adherence to the Greek Traditional Mediterranean Diet

The distribution of study participants according to sex, age, geographic region, and categories of Mediterranean diet score is shown in Table 5. Overall, 39.1% of participants were included in the intermediate category (score of 4–5 points) of the Mediterranean diet score, followed by 32.6% in the low (score 0–3 points) and 28.3% in the high (score of 6–9 points) categories. About 39.7% of participants over 65 years old and 25.5% of participants under 65 years old were included in the high category of the Mediterranean diet score. Of note, in the total HYDRIA cohort, 31.3% of men and 25.7% of women are characterized by a high adherence (score of 6–9 points) to the Mediterranean diet. Among geographic regions, individuals in Attiki had lower scores, with 35.6% and 25.7% of individuals included in the low and high categories of the Mediterranean diet, respectively, followed by the islands of Egeo and Kriti (33.1% in the low and 27.7% in the high category).

## 4. Discussion

The HYDRIA study is the first national health and nutrition survey conducted on a large representative sample of the adult population in Greece, providing evidence for the dietary and health status of the population [3]. Our results suggest that older Greeks were more adherent to the Greek traditional Mediterranean diet, consuming more vegetables, legumes, fruits, fish, and monounsaturated fats (primarily olive oil) than younger Greeks, who reported higher consumption of meat, mainly red meat, alcohol, cereals, saturated fats, and sugar products (particularly women). Regarding alcoholic beverages, older adults consumed more wine than beer, while younger ones consumed both equally. Individuals living in Attiki, followed by those in the islands of Egeo and Kriti, had lower adherence to the Greek traditional Mediterranean diet.

The above results are in line with previous findings from the Greek EPIC cohort [36] and the PA.ME.D.Y study [5]. In the PA.ME.DY study, younger individuals who were employed and had higher education were more adherent to a Western diet pattern characterized by the consumption of red meat, animal fats, and cheese. On the other hand, older participants who were less educated and lived outside of Attiki followed a diet closer to traditional dietary patterns, which included the consumption of fruit, vegetables, legumes, olive oil, seafood, and whole grains [5]. Findings from the Greek EPIC cohort indicated that younger adults were more likely to gradually abandon the Greek traditional Mediterranean diet, consuming more red meat and fewer fruits and legumes [36]. The current findings of the HYDRIA survey could explain the high prevalence of obesity, chronic diseases (diabetes mellitus, CVD), and their relevant risk factors (hypertension, high blood cholesterol, and glucose levels) observed among younger adults in Greece [3,4,9,10].

About one out of three adults (28.3%) was found to have high adherence to the Greek traditional Mediterranean diet. The percentage was lower among younger participants (25.5%) than the older ones (39.7%). These results could point to negative consequences in terms of morbidity and mortality among younger people. Specifically, several national [33,37,38,39,40] and international studies [41,42,43,44,45,46] suggested that lower adherence to traditional diets similar to the Mediterranean diet was associated with increased all-cause mortality and risk of various chronic diseases. The Greek EPIC cohort, which included 22,043 individuals, found an association between following the Greek traditional Mediterranean diet and a lower risk of all-cause mortality (HR:0.75, 95% CI: 0.64 to 0.87), death due to coronary heart disease (HR: 0.67, 95% CI: 0.47 to 0.94), and death due to cancer (HR:0.76, 95% CI: 0.59 to 0.98) [33]. Furthermore, a two-unit increase in the modified Mediterranean diet score was associated with a significant 8% reduction in overall mortality among older Europeans. At the same time, participants from Greece and Spain had a stronger association [46].

### 4.1. Comparison with Nutrient Reference Values

The higher energy intakes reported by the male population in the HYDRIA survey, which decreased with age, were consistent with those reported by other European national nutrition surveys [47,48]. The contribution of total fat in adults was found to be higher than the 20–35% dietary reference intake ranges (RI) suggested by EFSA [49]. Overall, total fat intake represents 42–43% of total energy intake in men and women in the HYDRIA survey, of which 20–22% of the total energy intake of individuals was represented by monounsaturated fatty acids. In addition, 25% of the older population, both men and women, had lower protein daily average intakes (51 g/day and 41 g/day) than the recommended daily average requirements for adults (AR), (57 g/day and 48 g/day), calculated based on EFSA’s recommendations [49]. It is of notice that the recommended daily protein intake is adequate for meeting the protein needs of 97.5% of the healthy adult population, as indicated by international bodies such as EFSA, WHO/FAO/UNU, IOM, DASH, and NNR, and is even higher (0.8–0.83 g/kg/bw/d) [50,51,52,53,54]. Moreover, the most recent recommended reference values for protein intake by DASH and NNR emphasize the higher protein needs of older adults (1 g/kg/bw/d and 15–20% of energy intake) and suggest a protein increase with decreasing energy intake. In the EPIC-PANACEA study, a higher percentage of energy from protein intake was associated with a 23–24% increased risk of being overweight or obese in normal weight and overweight individuals [45]. The above findings could be related to the high percentage (~70%) of overweight or obese adults in Greece, according to the preliminary results of the HYDRIA survey [3,4].

### 4.2. Comparison with Dietary Food Guidelines

**Figure 1:**
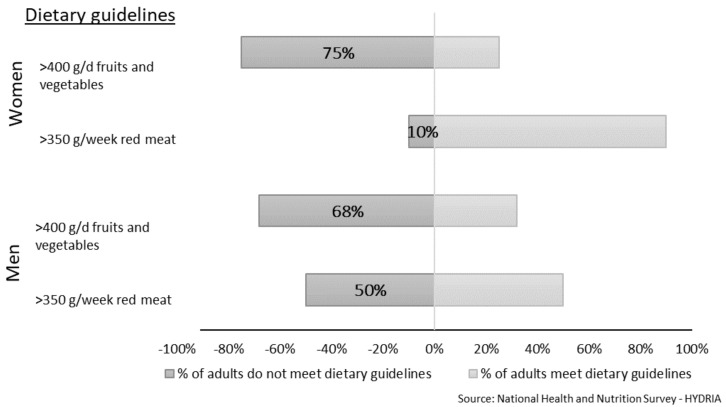
The percentage of adults meeting food dietary recommendations based on WHO and AICR/WRCF guidelines for fruits and vegetables (g/day) and red meat (g/week) in the Greek National Health and Nutrition Survey—HYDRIA.

To improve overall health and reduce the risk of cardiovascular diseases and certain types of cancer, WHO dietary guidelines recommend more than 400 g of fruit and vegetables per day [55] for adults, while AICR/WRCF recommends that red meat intake should not exceed 350–500 g per week [56]. Based on the percentile intakes found in the current study, the recommended amount of fruits and vegetables was not met by 68% of men and 75% of women in Greece, i.e., 32% of men and 25% of women consumed >400 g of fruits and vegetables per day (Figure 1). Men, especially younger men, did not follow the recommendations for red meat intake. About 50% of men overall (60% of men <65 years and 10% of men ≥65 years) ate more than 350 g (or 50 g/day), and 23% of men overall consumed more than 500 g of red meat per week (data for 500 g not shown in Figure 1). Among the factors that may influence these food choices are the globalization of the food supply, the urbanization of life, and general improvements in people’s socioeconomic conditions, which also lead to the increasing availability of animal-origin foods [57,58]. More and more international organizations are recommending limiting the consumption of meat and other sources of animal-protein foods and urging people to follow more environmentally friendly diets, which not only benefit health but are also associated with a lower ecological burden [55,59]. According to the EAT-Lancet Commission on healthy diets from sustainable food systems, the Mediterranean diet, which traditionally includes low consumption of animal products, is a diet that maximizes longevity, improves health-related quality of life, and at the same time is an ecologically sustainable and environmentally friendly way to eat [60]. The Greek traditional Mediterranean diet does not eliminate any food groups but rather encourages individuals to have a more plant-based diet. On the other hand, other diets that have gained popularity in recent years, such as the ketogenic diet, focus solely on restricting the percentages of macronutrients rather than their sources. Long-term evidence for the health benefits of such diets is weak, and low-carbohydrate and high-fat (in terms of saturated fats) diets have been related to increased mortality and cardiovascular diseases [61].

It is essential to mention that the HYDRIA survey implemented a harmonized protocol among EU Member States that generated data directly comparable to that of other European countries. Among the main strengths of the HYDRIA survey were the national representative sample and the standardized data collection. To collect dietary data, a specially designed dietary software application, the HHF Nutrition Tool, and a food composition table specifically designed for this project, the H-FCT, were used. The current study examined the usual intake of a wide range of food groups and selected subgroups. The differentiation between refined and whole grains in the cereal and products food groups was not possible for the current analysis. We only estimated the usual intake of adult men and women and not the dietary intake of children or adolescents, which was later collected in two additional studies. Overall, dietary and other health-related data were collected by interviewer-administered questionnaires. However, the HYDRIA survey has a cross-sectional design and thus cannot be used to prove causal relations, but it does contribute to identifying problems with a significant impact on the health of the population in the country.

## 5. Conclusions

In conclusion, the HYDRIA survey included a large national representative sample of the population in Greece that provided insights into the dietary behaviours of adults. Younger adults consume more meat, dairy, and alcohol, which are traditionally consumed at moderate-to-low levels in the Mediterranean diet, than fruit, legumes, and vegetables, which were consumed more by older individuals. Furthermore, adults did not meet international dietary recommendations for the consumption of red meat, fruit, and vegetables. Younger generations in Greece seem to move away from food choices included in the beneficial components of the Greek traditional Mediterranean diet at a time when other countries are modifying their diets to support the Mediterranean dietary pattern.

## Figures and Tables

**Table 1:** Selected sociodemographic and lifestyle characteristics † of participants in the Greek National Health and Nutrition Survey—HYDRIA.

	Men, %	Women, %
**Age**		
18–64 years	77.0	73.1
≥65 years	23.0	26.9
**Education level** ^1^		
Low	34.9	44.1
Intermediate	41.1	35.7
High	24.0	20.2
**Employment status** ^2^		
Employed	52.0	31.7
Unemployed	14.6	14.6
Students	5.4	5.0
Pensioners	28.0	22.1
Housewives	0.0	26.6
**Geographic area** ^3^		
Attiki	35.9	36.7
Northern Greece	28.4	28.6
Central Greece	25.5	24.6
Egeo and Kriti	10.2	10.1
**Physical activity** ^4^		
Sedentary	30.2	18.3
Light-intensity	66.1	80.5
Moderate-intensity	3.7	1.2
**Smoking status**		
Never smoker	30.3	54.8
Former smoker	30.3	12.8
Current smoker	39.4	32.4
**BMI** ^5^		
Obese	34.0	35.6
Overweight	43.4	31.0
Normal weight	22.3	31.1
Underweight	0.2	1.5

† Weighting factors were applied to allow for nationally representative results. ^1^ Education level was grouped into three categories: low, including illiterate individuals and those who had up to nine years of education/training; intermediate, including individuals who followed school (including any type of vocational training) for more than nine and up to twelve years; and high, including university graduates and postgraduate and/or doctoral degree holders. ^2^ Employment status was grouped in five categories: Employed individuals including unpaid helpers in family businesses, paid apprentices, investors, and persons temporarily absent from work due to sick leave, holiday leave, maternity or parental leave; those unemployed; students, including postgraduates and interns working without pay for the experience; pensioners; and housewives. ^3^ Geographic area was grouped into four categories: Attiki; Northern Greece, including the prefectures of Epirus, Eastern Macedonia, and Thrace; Western and Central Macedonia; Central Greece, including the prefectures of Central Greece, West Greece, Peloponnese, Ionian islands, and Thessaly; and Egeo and Kriti, including the Aegean islands and the island of Crete. ^4^ Physical activity, based on MET values grouped in three categories: 1.0–1.5 METs defined as sedentary behaviour, 1.6–2.9 METs defined as light-intensity, and 3–5.9 METs defined as moderate-intensity [28]. ^5^ The Body Mass Index (BMI) was calculated as body weight (in kilograms) divided by the square of height (in meters). Based on their BMI values, participants were grouped in four categories: obese: BMI ≥ 30 kg/m^2^, overweight: 25 ≤ BMI ≤ 29.9 kg/m^2^, normal weight: 18.5 ≤ BMI ≤ 24.9 kg/m^2^, and underweight: BMI < 18.5 kg/m^2^.

**Table 2:** Mean, median, and percentiles (25th and 75th) distribution † of the usual daily energy intake (kcal) in the Greek National Health and Nutrition Survey—HYDRIA.

	18+ Years				18–64 Years				65+ Years			
	Mean	p25	Median	p75	Mean	p25	Median	p75	Mean	p25	Median	p75
**Energy**												
Men	2192	1718	2109	2559	2331	1863	2237	2673	1727	1388	1683	2031
Women	1530	1221	1473	1763	1611	1304	1553	1838	1309	1057	1264	1502

† Mean energy intake was estimated using the mean of the two individual corresponding 24-HDRs under the assumption that two or more 24-HDRs per person give unbiased estimates of the single-day intakes. Median and percentile energy intake values were calculated using the National Cancer Institute (NCI) method [30,31,32]. Weighting factors were applied to allow for nationally representative results. Results include under-reporters.

**Table 3:** Energy percentage intake (%EI), median, and percentiles (25th and 75th) distribution (g) of the usual daily intake of macronutrients † in the Greek National Health and Nutrition Survey—HYDRIA.

Age Group	Men				Women			
	%EI	p25	Median	p75	%EI	p25	Median	p75
**Protein**								
18+ years	15.1	64	80	99	15.2	47	57	67
18–64 years	15.3	69	85	104	15.1	50	60	70
65+ years	15.1	51	64	78	15.3	41	49	58
**Glycaemic carbohydrates**								
18+ years	35.2	144	183	229	38.2	114	140	170
18–64 years	35.2	155	195	240	37.9	122	147	177
65+ years	35.0	116	147	183	38.2	100	121	146
**Fibre**								
18+ years	1.7	14	18	23	1.9	11	14	17
18–64 years	1.7	14	18	23	1.9	11	14	18
65+ years	2.0	13	17	21	2.2	11	13	17
**Total fat**								
18+ years	42.3	80	99	121	42.9	57	70	85
18–64 years	42.1	86	105	126	42.5	61	73	88
65+ years	43.3	65	80	98	43.3	49	60	72
**Saturated fatty acids, SFA**								
18+ years	13.1	24	31	38	13.5	18	23	28
18–64 years	13.1	26	33	40	13.4	18	24	29
65+ years	12.5	19	24	29	13.1	15	19	23
**Monounsaturated fatty acids, MUFA**								
18+ years	20.3	38	47	58	20.6	26	33	40
18–64 years	20.1	41	50	60	20.1	28	34	41
65+ years	21.9	32	40	49	21.3	23	29	36
**Polyunsaturated fatty acids, PUFA**								
18+ years	5.7	10	13	17	5.9	7	9	12
18–64 years	5.8	11	14	18	6.1	7	10	12
65+ years	5.7	8	11	13	5.5	6	8	10
**Alcohol**								
18+ years	5.4	3	10	23	1.8	0.6	2	5
18–64 years	5.7	3	10	25	2.2	0.9	3	6
65+ years	4.5	2	8	18	1.1	0.3	0.9	3

† Mean macronutrient intake was estimated using the mean of the two individual corresponding 24-HDRs under the assumption that two or more 24-HDRs per person give unbiased estimates of the single-day intakes. Median and percentile usual intake values were calculated using the National Cancer Institute (NCI) method [30,31,32]. Weighting factors were applied to allow for nationally representative results. Results include under-reporters.

**Table 4:** Mean (energy-adjusted), median, and percentiles (25th and 75th) distribution of the usual daily intake (g) of food groups and subgroups † in the Greek National Health and Nutrition Survey—HYDRIA.

Age Group	Men				Women			
	Mean	p25	Median	p75	Mean	p25	Median	p75
**Vegetables (without potatoes)**								
18+ years	198	147	203	270	217	116	158	206
18–64 years	186	146	202	268	204	115	157	204
65+ years	251	148	208	279	260	118	161	211
**Potatoes**								
18+ years	43	28	44	64	43	17	27	41
18–64 years	43	32	47	68	43	18	29	43
65+ years	42	21	33	49	43	15	24	36
**Legumes**								
18+ years	16	12	17	23	18	5	10	18
18–64 years	14	12	16	21	16	5	9	17
65+ years	25	17	22	29	23	6	11	20
**Fruit (without fruit juices)**								
18+ years	112	25	75	166	168	33	90	180
18–64 years	98	20	63	147	144	26	76	160
65+ years	177	50	119	224	249	61	133	223
**Cereals and products**								
18+ years	224	166	223	292	209	117	151	190
18–64 years	227	183	240	308	209	124	159	199
65+ years	211	127	169	223	211	102	131	164
**Bread**								
18+ years	127	98	134	175	110	60	78	97
18–64 years	118	103	141	183	110	63	81	100
65+ years	133	87	116	147	110	55	70	85
**Pasta**								
18+ years	53	28	47	73	48	31	39	48
18–64 years	51	34	53	79	48	34	42	50
65+ years	42	18	29	47	49	26	33	40
**Rice**								
18+ years	19	13	18	23	21	8	13	21
18–64 years	17	13	18	23	20	8	13	20
65+ years	22	11	16	21	26	9	14	22
**Dairy and products**								
18+ years	188	104	181	284	251	113	180	270
18–64 years	176	104	180	283	243	116	184	275
65+ years	240	103	182	285	281	105	169	256
**Milk**								
18+ years	112	23	82	188	162	37	96	189
18–64 years	103	22	79	185	154	37	96	190
65+ years	152	28	92	199	188	37	95	187
**Yoghurt**								
18+ years	22	2	7	24	38	2	18	40
18–64 years	17	1	6	21	36	2	18	39
65+ years	41	3	12	39	46	2	20	43
**Cheese**								
18+ years	49	12	50	74	47	13	37	50
18–64 years	47	15	55	79	50	16	40	53
65+ years	46	8	37	57	41	9	28	39
**Meat and products**								
18+ years	99	66	96	134	81	43	58	74
18–64 years	104	78	108	145	84	50	64	79
65+ years	73	42	61	85	70	31	43	56
**Poultry**								
18+ years	26	14	23	37	24	8	13	21
18–64 years	27	15	25	40	25	10	16	24
65+ years	21	10	17	27	17	5	8	13
**Red meat**								
18+ years	54	37	51	69	44	25	38	55
18–64 years	57	44	57	75	45	28	42	60
65+ years	42	27	34	42	44	19	28	40
**Fish (including seafood) and products**								
18+ years	24	8	16	34	26	8	14	26
18–64 years	21	7	16	32	22	7	13	23
65+ years	35	9	19	37	38	11	20	33
**Eggs**								
18+ years	15	8	13	21	16	6	10	15
18–64 years	15	9	14	23	15	7	11	16
65+ years	13	5	9	15	14	5	8	13
**Fats and oils**								
18+ years	44	35	44	56	44	25	32	40
18–64 years	42	36	46	57	43	25	32	41
65+ years	51	31	40	51	49	23	30	38
**Olive oil**								
18+ years	35	24	34	44	35	18	25	31
18–64 years	33	25	35	45	33	19	25	31
65+ years	43	23	32	42	40	18	24	30
**Nonalcoholic beverages**								
18+ years	124	46	104	243	121	26	55	117
18–64 years	136	62	136	302	137	37	73	145
65+ years	73	24	47	89	69	13	26	48
**Sugar and products**								
18+ years	26	11	22	39	39	13	24	41
18–64 years	27	14	25	43	42	16	28	47
65+ years	21	7	14	25	29	8	15	26
**Alcoholic beverages**								
18+ years	172	27	101	256	68	8	25	64
18–64 years	179	32	114	281	76	12	32	74
65+ years	133	14	68	178	40	3	12	34
**Wine**								
18+ years	80	5	23	95	34	4	11	28
18–64 years	78	6	23	95	36	5	13	31
65+ years	89	3	19	96	26	2	5	18
**Beer**								
18+ years	71	4	18	81	29	2	5	19
18–64 years	80	5	26	103	32	2	7	25
65+ years	32	1	5	25	12	1	3	8

† Mean food group and food subgroup intake were estimated using the mean of the two individual corresponding 24-HDRs under the assumption that two or more 24-HDRs per person give unbiased estimates of the single-day intakes. Usual mean intake of food groups and subgroups was energy-adjusted per 2000 kcal/day. Median and percentile usual intake values were calculated using the National Cancer Institute (NCI) method [30,31,32]. Weighting factors were applied to allow for nationally representative results. Results include under-reporters.

**Table 5:** Mediterranean Diet Score † in the Greek National Health and Nutrition Survey—HYDRIA.

	Low (Score of 0–3 Points)	Intermediate (Score of 4–5 Points)	High (Score of 6–9 Points)
**Total**, %	32.6	39.1	28.3
**Sex**, %			
Men	29.7	39.0	31.3
Women	35.1	39.2	25.7
**Age**, %			
<65 years	35.8	38.7	25.5
≥65 years	19.5	40.8	39.7
**Geographic area** ^1^, %			
Attiki	35.6	38.7	25.7
Northern Greece	30.0	39.7	30.3
Central Greece	31.2	39.0	29.7
Egeo and Kriti	33.1	39.2	27.7

† The Mediterranean diet score was calculated using the National Cancer Institute (NCI) method [34,35]. Weighting factors were applied to allow for nationally representative results. Results include under-reporters. ^1^ Geographic area was grouped into four categories: Attiki; Northern Greece including the prefectures of Epirus, Eastern Macedonia and Thrace; Western and Central Macedonia; Central Greece including the prefectures of Central Greece, West Greece, Peloponnese, Ionian islands and Thessaly; Egeo and Kriti including the Aegean islands and the island of Crete.

## Data Availability

Not additional data available.
